# Supplementary material for: Induction of Senescence by Loss of Gata4 in Cardiac Fibroblasts
Source: Cells. 2023 Jun 17;12(12):1652. doi: 10.3390/cells12121652 (PMC10297635; doi:10.3390/cells12121652)
Supplement: Supplementary file 1 [file cells-12-01652-s001.zip › cells-2394904-supplementary.pdf]

Supplementary Materials

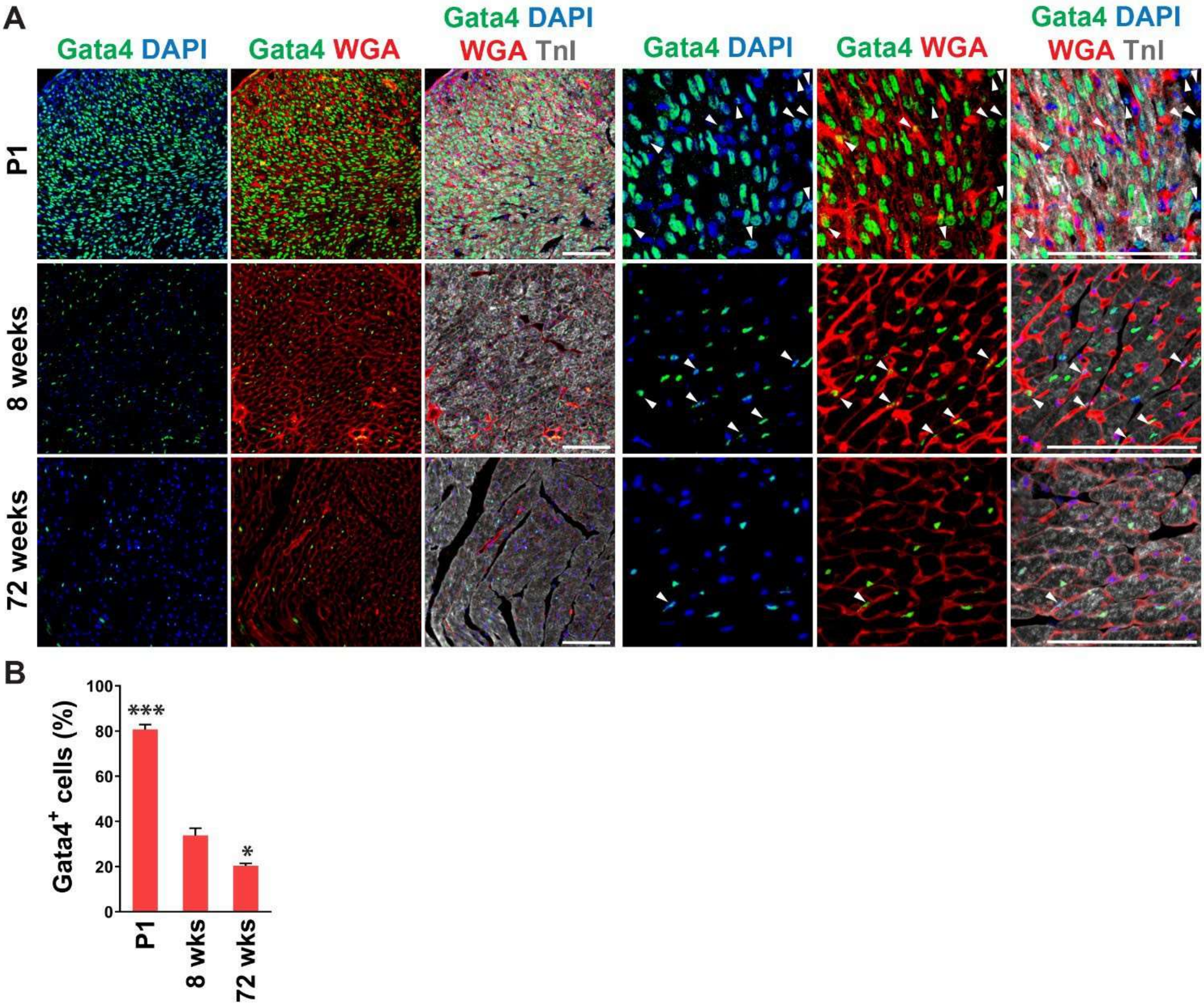

Supplementary Figure S1. Temporal expression pattern of Gata4 in the heart. (**A**) The number of Gata4-expressing cells decreases in the aged heart. Heart sections from different ages of mice, including P1, 8 weeks, and 72 weeks old, were immunostained for Gata4 and Troponin I (TnI), along with wheat germ agglutinin (WGA) staining to demarcate cell boundaries. White arrowheads indicate Gata4$^+$ interstitial cells in WGA stained interstitial area. Gata4$^+$Troponin I$^+$ cells surrounded by WGA staining indicate Gata4$^+$ cardiomyocytes. Scale bar, 100 μM. (**B**) Quantification of Gata4$^+$ cells in the hearts of different ages. Three independent experiments are presented as mean+s.d. $n = 3$, * $p < 0.05$; *** $p < 0.0001$ vs. 8 weeks. wks: weeks.

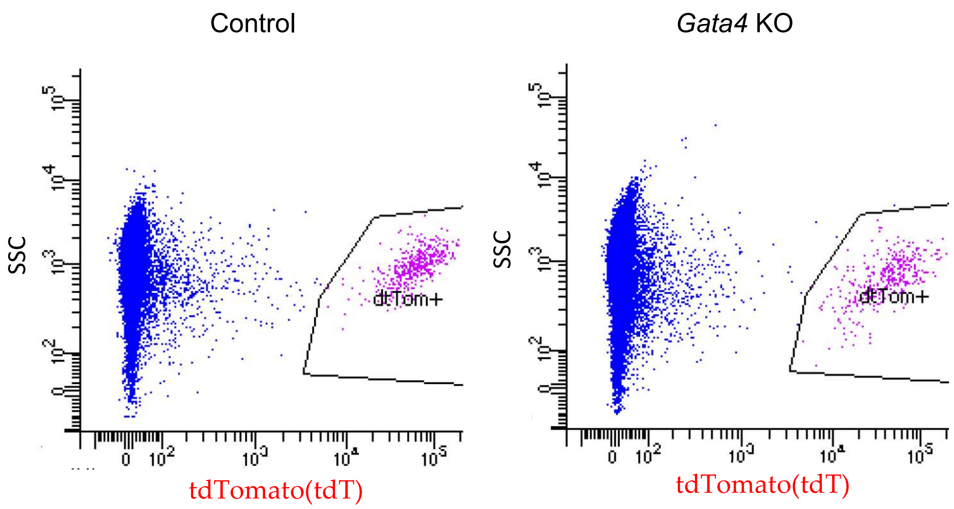

Supplementary Figure S2. Non-myocytes from *Tcf21iCre:R26RtdT* (Control) and *Tcf21iCre:Gata4fl/fl:R26RtdT(Gata4* KO*)* mice. A week after administering tamoxifen into *Tcf21*^{iCre}*:R26R*^{tdT} (Control) and *Tcf21*^{iCre}*:Gata4*$^{fl/fl}$*:R26R*^{tdT} (*Gata4* KO) mice, non-myocytes isolated and sorted out tdTomato^{+} cells using fluorescence-activated cell sorting(FACS).

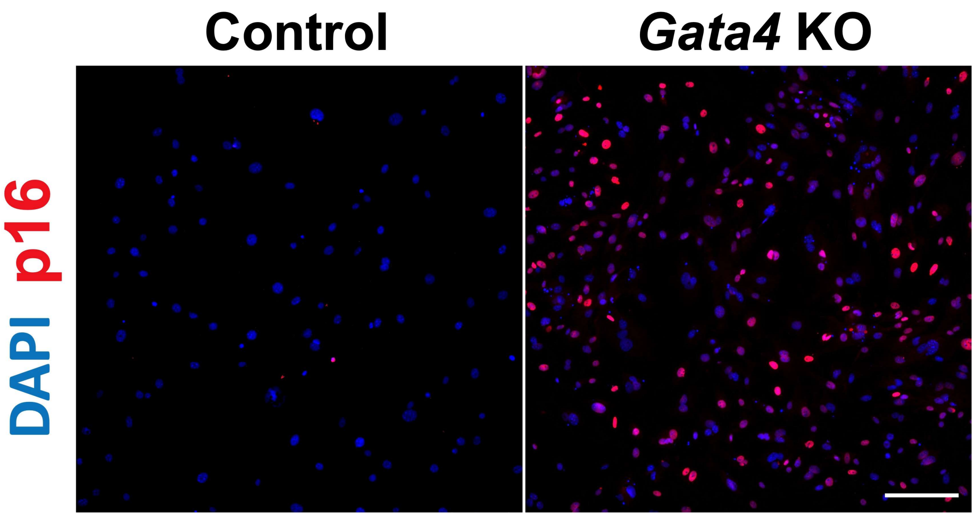

Supplementary Figure S3. Senescence induction in Gata4 deficient cardiac fibroblasts in vitro. Demonstration of senescence in Gata4 deficient cardiac fibroblasts. *Gata4*$^{fl/fl}$ cardiac fibroblasts infected with Ad-vector (Control) or Ad-Cre (*Gata4* KO) were immunostained for p16. Scale bar, 100 μM.

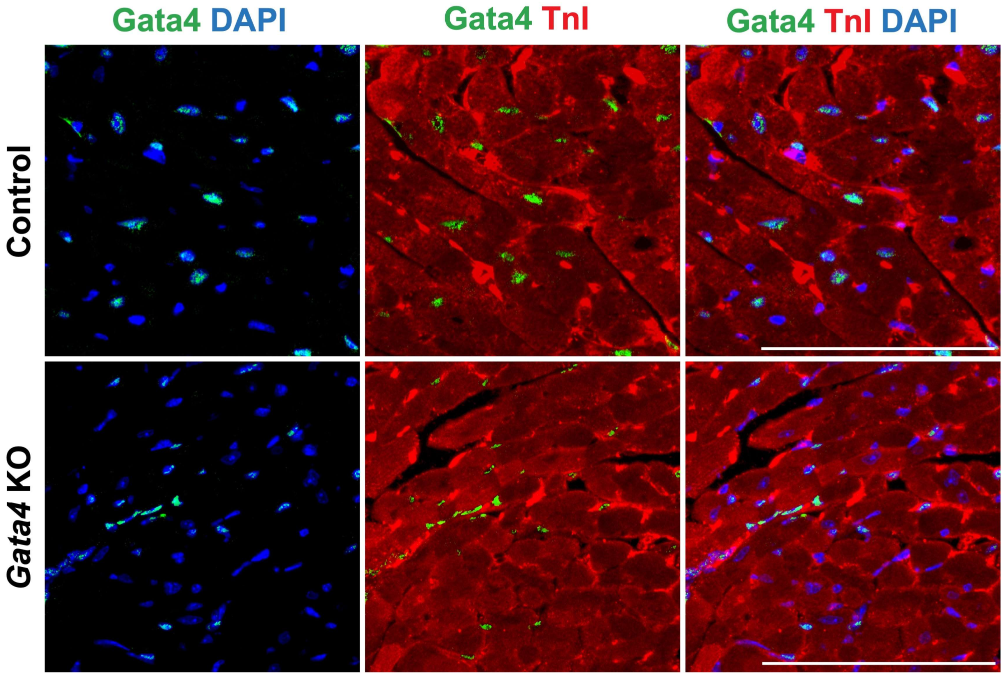

Supplementary Figure S4. Generation of cardiomyocyte-specific, inducible Gata4 KO mice. Immunofluorescent analysis to demonstrate Gata4 inactivation in *αMHC* lineage traced cardiomyocytes. A week after tamoxifen administration, αMHC^{iCre} (Control) and $\alpha MHC^{iCre}{:}Gata4^{fl/fl}$ (*Gata4* KO) mouse hearts were harvested, and heart sections were processed. The heart sections were immunostained for Gata4 and stained for Troponin I (TnI) to demarcate Cardiomyocyte. White arrowheads indicate $Gata4^{+}$ cardiomyocytes. Scale bar, 100 μM.

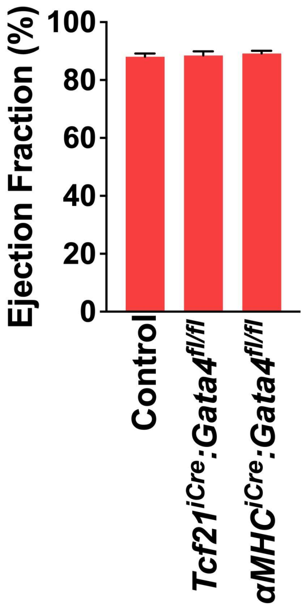

Supplementary Figure S5. *Tcf21^{iCre}: Gata4$^{fl/fl}$*, and *αMHC^{iCre}:Gata4$^{fl/fl}$* and WT mice were examined by transthoracic echocardiogram. One week after administering tamoxifen into *Tcf21^{iCre}:Gata4$^{fl/fl}$* and *αMHC^{iCre}:Gata4$^{fl/fl}$* and WT (Control) mice, ejection fraction was tested by using a transthoracic echocardiogram. Three independent experiments are presented as mean ± s.d. $n = 3$.

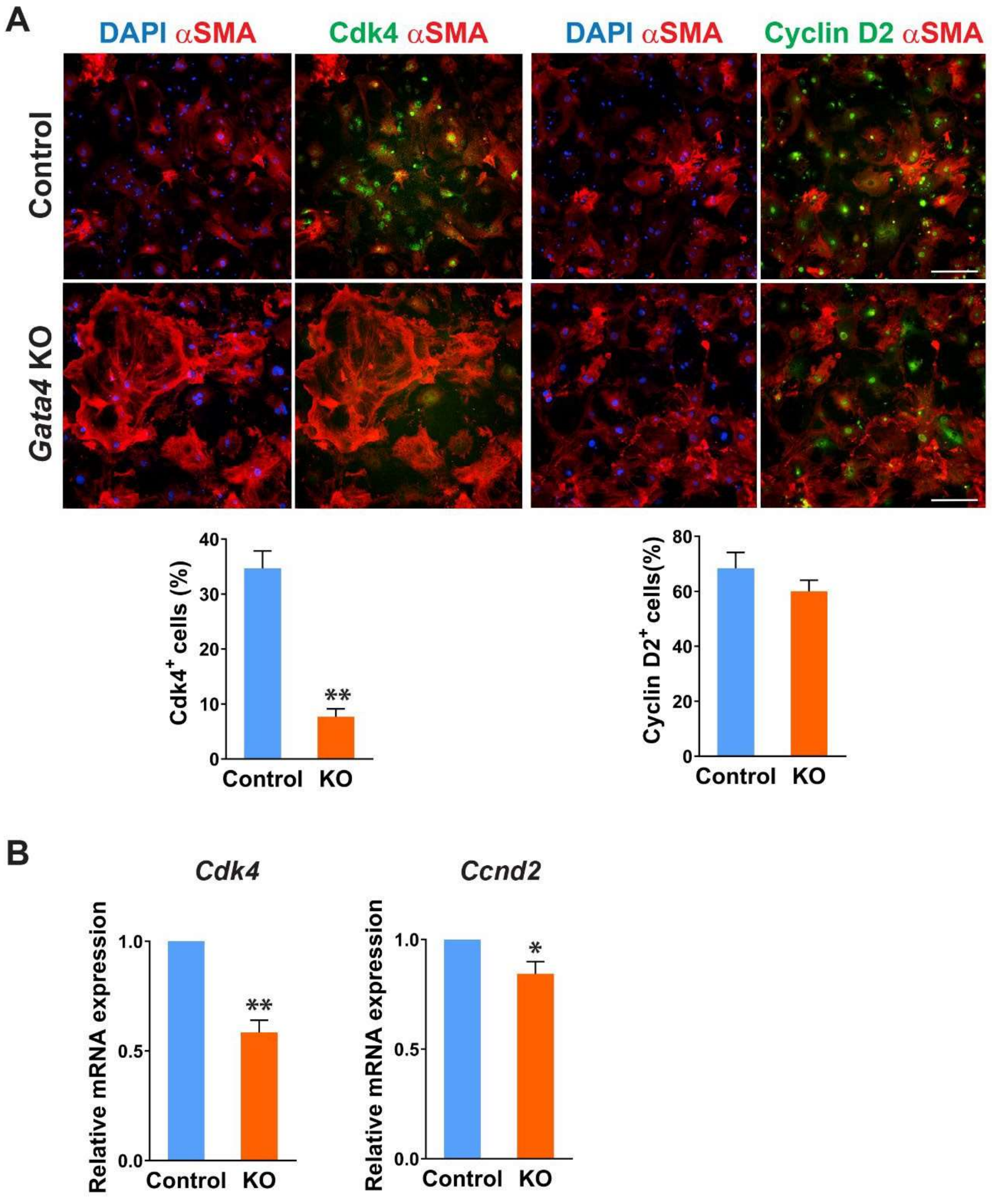

Supplementary Figure S6. Assessing expression levels of Cdk4 and Cyclin D2 following the loss of Gata4 in cardiac fibroblasts. (**A**) Ad-Cre or Ad-vector viruses were infected into cardiac fibroblasts isolated from $Gata4^{fl/fl}$ mice. Ten days later, the infected cells were immunostained for SMA and Cdk4 or Cyclin D2. Representative immunofluorescent images of cells infected with Ad-vector (control) or Ad-Cre (*Gata4* KO) were shown. $Cdk4^{+}$ or Cyclin $D2^{+}$ cells were quantified using high-content imaging analysis. Three independent experiments are presented as mean ± s.d. $n = 3$, ** $p < 0.005$. Scale bar, 200 μM. (**B**) qPCR analysis for mRNA levels of *Cdk4* and *Ccnd2* using sorted $tdTomato^{+}$ cells from $Tcf21^{iCre}$:$R26R^{tdT}$ (control) and $Tcf21^{iCre}$:$Gata4^{fl/fl}$:$R26R^{tdT}$ (KO) mice. Three independent experiments are presented as mean ± s.d. $n = 3$, * $p < 0.05$; $n = 3$, ** $p < 0.005$.

Supplementary Table S1. Sequence of primers used for RT-PCR studies.

Gene	Forward Primer (5′–3′)	Reverse Primer (5′–3′)
Gata4	GCCGAGGGAGCCGCCTACAC	TGGGGTGTCTTCCAGGGTTGG
Cdk4	ATGGCTGCCACTCGATATGAA	TCCTCCATTAGGAACTCTCACAC
Ccnd2	GAGTGGGAACTGGTAGTGTTG	CGCACAGAGCGATGAAGGT
GAPDH	CTCCCACTCTTCCACCTTCG	CCACCACCCTGTTGCTGTAG
